# Effect of Outflow Tract Banding on Embryonic Cardiac Hemodynamics

**DOI:** 10.3390/jcdd3010001

**Published:** 2015-12-24

**Authors:** Venkat Keshav Chivukula, Sevan Goenezen, Aiping Liu, Sandra Rugonyi

**Affiliations:** 1 Department of Mechanical Engineering, University of Washington, Stevens Way, Box 352600, Seattle, WA 98195, USA; cvkeshav@u.washington.edu; 2 Department of Mechanical Engineering, Texas A&M University, College Station, TX 77840, USA; sgoenezen@tamu.edu; 3 Department of Biomedical Engineering, University of Wisconsin-Madison, 1550 Engineering Drive, ECB 2145, Madison, WI 53706, USA; aliu26@wisc.edu; 4 Department of Biomedical Engineering, Oregon Health & Science University, 3303 SW Bond Ave. M/C CH13B, Portland, OR 97239, USA; rugonyis@ohsu.edu

**Keywords:** chicken embryonic heart, outflow tract, computational fluid dynamics (CFD), congenital heart disease, cardiovascular development, hemodynamics, cardiac development

## Abstract

We analyzed heart wall motion and blood flow dynamics in chicken embryos using *in vivo* optical coherence tomography (OCT) imaging and computational fluid dynamics (CFD) embryo-specific modeling. We focused on the heart outflow tract (OFT) region of day 3 embryos, and compared normal (control) conditions to conditions after performing an OFT banding intervention, which alters hemodynamics in the embryonic heart and vasculature. We found that hemodynamics and cardiac wall motion in the OFT are affected by banding in ways that might not be intuitive *a priori*. In addition to the expected increase in ventricular blood pressure, and increase blood flow velocity and, thus, wall shear stress (WSS) at the band site, the characteristic peristaltic-like motion of the OFT was altered, further affecting flow and WSS. Myocardial contractility, however, was affected only close to the band site due to the physical restriction on wall motion imposed by the band. WSS were heterogeneously distributed in both normal and banded OFTs. Our results show how banding affects cardiac mechanics and can lead, in the future, to a better understanding of mechanisms by which altered blood flow conditions affect cardiac development leading to congenital heart disease.

## 1. Introduction

During embryonic development, the heart transforms from a linear tube to a four-chambered heart. Cardiogenesis is tightly controlled by genetic programs, but it is well known that pre-programmed events are modulated by blood flow conditions [1,2,3,4,5]. Because the heart starts beating soon after formation of the linear tube, essential cardiac morphogenesis processes occur under blood flow conditions. Normal blood flow is essential for proper heart development, and altered blood flow can lead to detrimental morphogenesis and the formation of heart defects [1,4,6,7,8,9]. Thus, the study of cardiac development is intrinsically linked to the study of cardiac function and cardiac mechanics over embryonic and fetal developmental stages.

To fully understand cardiogenesis, assessment and quantification of embryonic cardiac dynamics (cardiac hemodynamics and cardiac wall motion) is required. However, studies are hampered by difficulties in imaging the fast cardiac beating dynamics, and measuring blood pressure and blood flows in the tiny developing heart. For mammalian models, these difficulties are compounded by accessibility of the embryos inside the mother’s womb. Not surprisingly, studies focusing on embryonic cardiac dynamics frequently use the chick or the zebra fish models [3,9,10]. Emerging techniques that have been used to study the chick or zebra fish embryos are, however, lately being applied to mice models as well [11,12]. In this manuscript we describe our strategy to study and quantify the chick embryonic cardiac dynamics under normal and altered blood flow conditions.

Our approach to study cardiac dynamics uses imaging and computational modeling techniques. We focus on the chick heart at early developmental stages (three days of incubation out of 21 days to hatching, which corresponds to approximately four weeks of gestation in a human baby). At these stages, the heart is tubular and sensitive to hemodynamic insults that could result in altered development of valves and chambers. We further focus on the heart outflow tract (OFT), the distal portion of the heart that connects it to the circulation, and that later gives rise to a portion of the interventricular septum, the semilunar valves, and the pulmonary and aortic trunks.

To quantify cardiac dynamics, we need to accurately image the fast beating embryonic heart (~2.5 Hz), and blood flow velocities within the heart. Optical coherence tomography (OCT) is a relatively recent optical imaging technology that allows tomographic imaging of the chick heart with high resolution (~5 µm) enabling microstructural cardiac imaging [13,14,15,16,17,18]. Early during development, the tubular heart consists of three layers: myocardium, cardiac jelly, and endocardium (at the lumen-wall interface); and these three layers can be distinguished with OCT. Since no contact is necessary, fast acquisition OCT systems are ideal for studying embryonic heart motion *in vivo*. In addition, because OCT simultaneously acquires structural and Doppler velocity data, OCT cardiac imaging can be used to determine both cardiac tissue motion and blood flow dynamics [19,20,21]. We have developed strategies to reconstruct 4D images (3D + time) of the beating heart using OCT [22], and to segment the heart layers over time [23], enabling analysis of heart motion under normal and altered hemodynamic conditions. Further, we have developed a computational fluid dynamics (CFD) model strategy to quantify embryo-specific blood flow conditions in the heart OFT [24]. CFD modeling was used to complement imaging data in capturing the details of blood flow within the OFT, and in facilitating computation of wall shear stresses, a recognized stimulant of mechanotransduction leading to tissue remodeling [2,25,26,27,28]. Using these techniques, we analyzed blood flow and cardiac motion of the day 3 embryonic chick heart OFT under normal conditions and after altering hemodynamics by outflow tract banding, an intervention in which a suture restricts OFT cardiac motion affecting hemodynamics. Outflow tract banding is a well-established hemodynamic intervention that is known to lead to a spectrum of congenital heart defects later during gestation [8,9]. Moreover, recent studies have started to identify changes in gene expression affecting early tissue remodeling in banded hearts [29]. We found differences among control and banded embryos not only in blood flow conditions but also on the dynamics of the OFT beating tissues. These cardiac mechanical changes contribute to the formation of heart defects and are intrinsically linked to congenital heart disease.

## 2. Methods

We describe our approach to capture and quantify wall motion and hemodynamics in the OFT of chicken embryos under normal and altered blood flow conditions. The methods are based on OCT images, from which we also construct embryo-specific computational hemodynamic models of blood flow dynamics.

### 2.1. Embryo Preparation and Hemodynamic Intervention

Embryo preparation and OCT imaging were described in detail in our earlier work [22,30]. Briefly, we placed fertilized white leghorn eggs into an incubator (Genisys 1588, Savannah, GA, USA) blunt-end up at 38 °C and 85% humidity for about 72 h. Embryos were then removed from the incubator, a small window was opened on the egg shell, and embryonic stages checked following Hamburger and Hamilton staging [31]. To expose the heart, the underlying membrane was removed. Since temperature affects cardiac function, during manipulation and imaging acquisition the temperature of chicken embryos was maintained at 37.5 ± 0.5 °C within a custom-made warming chamber and a temperature controller.

Two groups of embryos were studied: (1) a normal control group (*n* = 7); and (2) an outflow-tract banded (OTB) group (*n* = 6). For the normal control group, no further procedures were performed. For the banded group, a surgical suture (10-0 nylon suture) was passed around the OFT inlet region and tied with a knot. Placement of the suture (band) partially constricted the luminal area at the banding site. Immediately after banding, the egg shell was resealed with parafilm and re-incubated for an hour before OCT imaging was performed. Supplementary videos S1 and S2 show, respectively, a beating normal and banded chick heart at day 3 of incubation (HH18).

### 2.2. OCT Imaging

OCT images were acquired using our custom-made system, optimized to image the structure of the chick OFT *in ovo* [17,22]. This OCT system (central wave length λ = 1310 nm; line scan rate 47 kHz) yielded an axial spatial resolution of 10 µm and a lateral spatial resolution of 16 µm. We acquired *in vivo* images of the OFT consisting of 512 × 256 pixels (256 A-scans) at 140 frames per second. OCT is a non-contact imaging technique, and thus to image the chick embryonic heart OFT, we placed the windowed egg inside a temperature-controlled chamber, and under the OCT beam for imaging. Because at early stages of incubation (HH18) the embryo is on top of the yolk with its heart showing on top of its body, the heart could be easily localized with this setup.

To capture the dynamics of the beating embryonic heart (typical heart rate is 2.5 Hz at HH18), we used a 4D imaging strategy and reconstruction procedure [22]. Namely, we acquired sequences of cross-sectional frames over the length of the OFT that were then reconstructed into 3D images over time. Briefly, our imaging protocol consisted of imaging a single 2D OFT cross-section over time (B-mode) for a total of 195 frames (about 4 cardiac cycles or heartbeats), and then repeat the B-mode acquisition at another (consecutive) cross-section. Following this procedure, we acquired 65 B-mode cross-sectional images along the OFT (proximally to distally) with 12.5 µm distance between consecutive cross-sections. A B-mode of a longitudinal section was also acquired for post-processing synchronization. Data acquisition was non-gated; thus we synchronized the images making use of the periodicity of the cardiac cycle, structural similarity, and the longitudinal B-mode image sequence, to capture the peristaltic-like motion of the developing heart OFT [5]. Our post-processing image synchronization strategy allowed us to obtain 4D (3D + time) structural images of the embryonic heart OFT.

OCT signals, in addition, were used to measure the phase shift between line scans, Δϕ, from which velocity data were extracted. This is an important feature of OCT imaging, since it enables processing of flow velocity data from OCT data, resulting in velocity data for each individual imaging structural pixel. Doppler velocity was computed from Δϕ as follows [20,32],

(1) $$V_{z}=\frac{\lambda \;\Delta \phi }{4\;\pi \;n\;\tau }$$

where $V_{z}$, the Doppler velocity, is the vertical component of the velocity (the direction of the OCT light beam), and for our system, the refractive index was *n* = 1.35, and the time difference between two adjacent line scans (1/A-scan rate) was τ = 21 µs. The Doppler feature of OCT was essential to simultaneously obtain structural and hemodynamic data with the same pixel resolution from the *in vivo* images of the embryonic heart OFT.

### 2.3. OCT Image Processing

#### 2.3.1. Cross-Sectional Analysis

**Figure 1:**
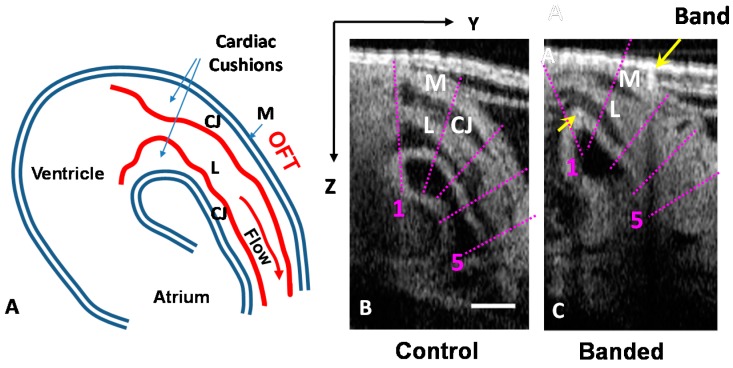
OCT images of the chick heart OFT. (**A**) Schematic of chicken heart at HH18. Note that details of the lumen-wall interface (red line) are given only for the OFT portion of the heart; (**B**) Longitudinal section of a normal OFT during OFT myocardial contraction; (**C**) Longitudinal section of a banded OFT, yellow arrows pointing at the band. Overlaid purple lines show the locations (1 to 5) from which cross-sectional images were extracted from the 4D OCT images for further analysis. Scale bar = 200 µm. M: Myocardium; L: Lumen; CJ: Cardiac jelly, with cardiac cushions being thickenings of the CJ.

To extract the dynamics of OFT wall motion from the 4D OCT images we first analyzed cross-sectional images over time. To this end, we developed and implemented a set of image-analyses algorithms using Matlab (MathWorks, Inc., Natick, MA, USA). From each 4D image set we selected a 3D image featuring the OFT in its most contracted phase. We segmented the myocardial wall and calculated the OFT centerline from this 3D image. We then selected 5 evenly-spaced points along the centerline, in such a way that points from different embryos were approximately corresponding. Using the 4D image data set, we then extracted 2D cross-sectional image sequences, such that image planes were centered at the 5 selected points and perpendicular to the centerline (see Figure 1, with cross-sections represented by lines 1 to 5). From the extracted cross-sectional image sequences, we delineated the boundaries of the OFT myocardium and lumen frame-by-frame during the cardiac cycle, and calculated the areas and perimeters of the OFT wall regions for further analysis.

Deformation patterns of the OFT wall were characterized by the cross-sectional area shortening fraction (*ASF*) and circumferential strain. *ASF* measures the total change in area relative to the maximum area:

(2) $$ASF=\frac{A_{\max}-A_{\min}}{A_{\max}}$$

where *A*_max_ and *A*_min_ are, respectively, the maximum and minimum areas of the lumen or myocardium. Here, the area of the myocardium is computed as the area enclosed by the inner myocardium contour.

The circumferential strain of the endocardium or myocardium was estimated by:

(3) $$\varepsilon_{\theta }=\frac{\left|C-C_{\max}\right|}{C_{\max}}$$

where *C* is the perimeter of the inner myocardium contour or the lumen-wall contour, calculated from image segmentation. The circumferential strain was estimated using Equation (3), which used the maximal perimeter of the OFT contours as the reference (zero strain), thus the circumferential strains computed were compressive and reflected the contraction of the myocardium or endocardium.

To quantify the valve-like behavior of the OFT, we extracted M-mode images along a line containing the OFT cushions (perpendicular to the slit-like lumen during lumen closure) when the OFT was most contracted. Endocardial cushions are thickenings of the cardiac jelly towards the lumen that facilitate lumen closure. From the extracted M-mode images, we identified and quantified the portion of the cardiac cycle during which the cardiac wall was contracting and expanding, and the fraction of time the lumen was closed. We also estimated the total fraction of the cardiac cycle during which the entire OFT lumen was closed, defined as the total time that at least one cross-section featured a closed lumen.

#### 2.3.2. 4D Segmentation

We used an in-house OCT image segmentation procedure, described in detail previously [23], to extract the dynamics of OFT wall motion from 4D OCT images. Briefly, the algorithm allowed extraction of OFT lumen and myocardium surfaces as a sequence of meshes over time during the cardiac cycle. To improve analysis of wall motion and facilitate volumetric mesh generation for CFD modeling, we implemented a consistent mesh parameterization strategy followed by a strain minimization algorithm [33,34]. The parameterization discretized the tubular lumen and myocardium surfaces using a cylindrical-like approach: with quasi cross-sectional planes intersecting the surfaces and defining contours (“rings”) along the OFT that were in turn uniformly discretized. This resulted in structured meshes formed by “rings” along the tubular OFT structures that defined cross-sectional planes. These structured meshes were employed for visualizing the dynamic motion of the OFT walls and to develop computational hemodynamic models of the OFT.

To visualize cardiac OFT wall motion, we used 2D area plots extracted from structured surface meshes. These plots display the normalized cardiac cycle time in the x-direction and normalized OFT segmented length in the y-direction. Lumen areas or myocardial areas, which are the areas enclosed by the lumen and myocardium mesh contours, respectively, were then color-coded in the 2D area plot, so that changes in area over time and along the OFT tube can be visualized. 2D area plots showed changes in OFT wall motion due to normal biological variations (*n* = 4) and banding (*n* = 4).

The parameterized surface meshes were then used to construct structured volume meshes of the OFT lumen that captured the motion of the OFT over time. These volume meshes were used in embryo-specific CFD models of the heart OFT under normal (*n* = 1) and banded (*n* = 1) hemodynamic conditions.

### 2.4. 4D CFD Embryo-Specific Modeling of the Heart Outflow Tract

We chose one representative normal (control) embryo and one representative banded embryo for 4D CFD embryo-specific modeling of the OFT. The CFD models were used to capture the details of blood flow within the OFT and to facilitate computation of wall shear stresses. Thus, CFD models complemented OCT imaging and velocity data. This is because Doppler OCT only measures one component of the 3D velocity vector (the component in the direction of the OCT beam) and limitations in Doppler velocity acquisition make Doppler data noisy and inaccurate in several portions of the OFT. Our CFD models of the heart OFT used lumen geometries and blood flow velocities extracted from 4D OCT images, as described in detail previously [24]. Briefly, an optimization procedure was employed in the CFD simulations to determine embryo-specific blood flow velocities within the OFT. To this end, for each time step simulated we used the lumen mesh (obtained by segmenting the OCT images) and velocity data from one point within the lumen (extracted from corresponding Doppler OCT images). The lumen geometry and point-velocity data were used in an iterative, inverse-method algorithm, which optimized pressure boundary conditions (more precisely inlet pressure minus outlet pressure, or the pressure drop Δp) such that the difference between CFD computed and Doppler OCT measured velocity at the selected point was minimized. The optimization procedure was implemented using in-house developed programs on a high performance cluster (HPC) available at Oregon Health and Science University (OHSU). Use of our optimization procedure ensured that both the geometry and velocity profiles within the lumen were embryo-specific, which is particularly important for accurately simulating hemodynamic conditions after the banding intervention.

The dynamics of blood flow within the OFT over the cardiac cycle was simulated using quasi-steady assumptions, thus neglecting inertial effects. This approximation is justified because flow in the small developing OFT is dominated by viscous effects (with inertia being negligible) as reflected by the small Reynolds (Re < 7) and Womersley (Wo < 0.5) numbers. At each simulated time over the cardiac cycle (time step), blood flow was modeled using the Navier-Stokes equations (with blood density ρ = 1060 kg/m^3^, and blood viscosity μ = 0.003 kg/(m s)), and no-slip boundary conditions at the lumen-wall interface (which neglects wall velocities). The OFT wall motion, however, was accounted by incorporating the changing lumen geometry over time. This strategy generated accurate results except when blood flow velocities were small, *i.e.*, the OFT lumen was just starting to open or near closure [24,30], since at those times wall velocities were not negligible in comparison to flow velocities. Further, since blood flows through the OFT only during approximately half of the cardiac cycle [21,30], we modeled 51% and 40% of the normal and banded embryo cardiac cycles, respectively. CFD simulations were performed using ADINA (Watertown, MA, USA), with the lumen volume meshed using 8-node flow-condition-based interpolation (FCBI) elements. Prior to simulating the models, a convergence study was performed to determine accuracy and convergence of solutions.

## 3. Results and Discussion

We characterized the wall motion and hemodynamics in normal and banded embryos at stage HH18, and analyzed changes introduced by a banding hemodynamic intervention.

### 3.1. OFT Wall Motion: Cross-Sectional Analysis

**Figure 2:**
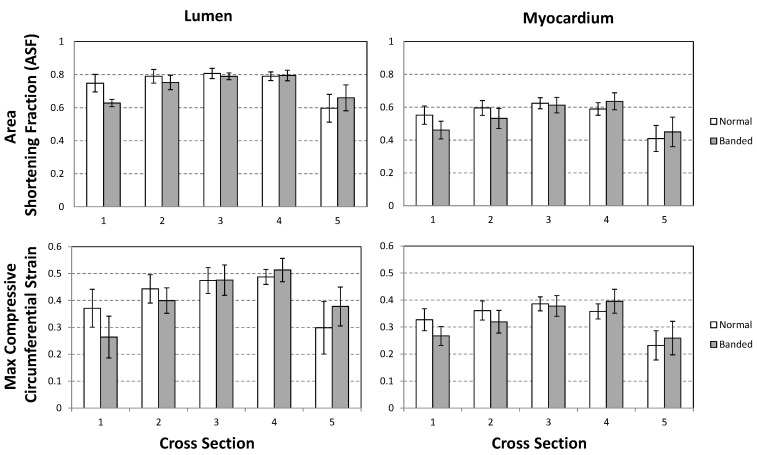
Comparison of OFT wall motion of control and banded embryos. Analysis was done based on 4D OCT images (control *n* = 7; banded *n* = 6), with cross-sectional locations indicated in Figure 1. The data compares *ASF* on top (Equation (2)) and maximum compressive circumferential strain on bottom (Equation (3)) for the lumen (**left**) and myocardium (**right**). Data displays average ± SD.

The effects of banding on OFT wall motion were apparent by examining the expansion and contraction of the lumen and myocardium from cross-sectional views (see Figure 1, with the band typically placed somewhere between cross-sections 1 and 3). The maximal lumen area in banded hearts was reduced around the band site, as expected. On average, *ASF* of the lumen and myocardium, a measure of cardiac wall contractility and function, only reduced around cross-section 1 (see Figure 2), indicating the effect of the band that limited cardiac expansion.

Circumferential strains in the myocardium and endocardium of control and banded embryos varied along the OFT (see Figure 2). Largest compressive circumferential strains occurred during maximal OFT contraction. Maximum compressive circumferential strains in the endocardium were larger than those in the myocardium at corresponding cross-sections. Compared to control, the circumferential strain of banded embryonic hearts significantly decreased in the myocardium and the endocardium proximally (upstream) to the band (cross-section 1; see Figure 2). Maximum compressive circumferential strain, a measure of myocardium contractility, recovered its normal values downstream of the band. Overall, these data suggest that myocardial function is only restricted at the band site (due to band constriction). *ASF* and circumferential strains do not seem affected by the reported increased blood pressure [35] downstream of the band.

In addition to the effects of banding on OFT wall motion, two characteristics of the OFT dynamics emerged from the analysis in Figure 2. First, myocardium motion (and thus likely contractility) reduced towards the downstream end of the OFT. This is not entirely surprising, as the active OFT myocardium merges downstream with the aortic sac, which does not exhibit active contraction. Second, the dynamic contraction and expansion of the myocardium was “amplified” in the lumen, which exhibits larger variations in area (*ASF*) and circumferential strain. This characteristic is mainly due to the influence of endocardial cushions, which are thickenings of the cardiac wall towards the lumen, and that are located on two opposite sides of the OFT wall. Due to the endocardial cushion placement, when the OFT walls are fully expanded the lumen has an almost perfectly circular shape, but as the walls contract, the lumen shape becomes more elliptical, until the lumen closes forming a slit-like shape. In allowing the lumen to fully close (with opposite endocardial cushions coming in contact) and fully open (with endocardial cushion expanding to very thin layers), the endocardial cushions function as primitive valves that prevent backflow at HH18 (see also [36]).

**Figure 3:**
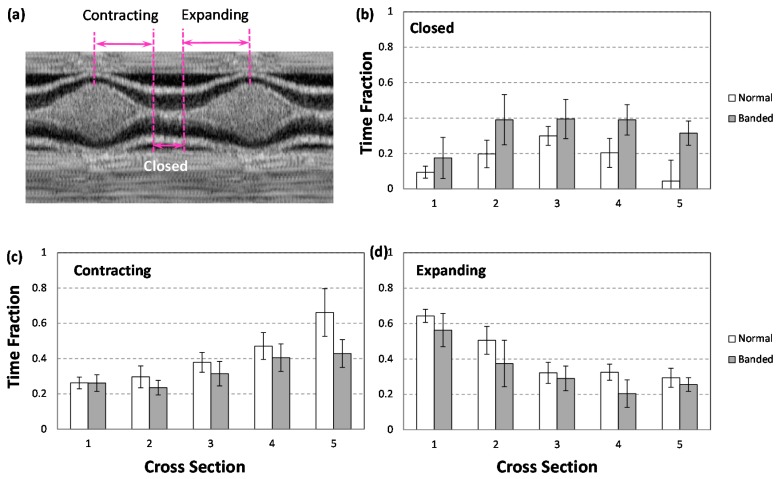
OFT wall dynamics under control and banded conditions. Analysis was done based on 4D OCT images (control *n* = 7; banded *n* = 6), with cross-sectional locations indicated in Figure 1. (**a**) shows an M-mode OCT image to illustrate the criteria employed to describe the wall motion. The data shows comparisons of the fraction of the cardiac cycle over which (**b**) the lumen was closed and OFT walls were (**c**) contracting and (**d**) expanding. Data displays average ± SD.

To further determine changes in the OFT wall dynamics in banded embryos, we analyzed cyclic wall motion characteristics. To this end we used M-mode images extracted from the five selected OFT cross-sections, and determined the percentages of the cardiac cycle during which the walls were expanding and contracting, and the lumen was closed (see Figure 3). In control embryos, the fraction of time during the cardiac cycle the OFT walls were expanding decreased proximally to distally, whereas the contraction time increased proximally to distally. The same trend remained in banded embryos, but with reductions at the distal portion of the OFT (cross-sections 4 and 5). The fraction of time the OFT lumen was closed changed in banded embryos relative to normal controls. We found that the fraction of the time at which the lumen was closed significantly increased in banded embryos at cross-sections 2, 4 and 5, even though total closure time of the entire OFT remained unchanged (~0.5 T, with T the cardiac cycle period).

Interestingly, the band affected the downstream dynamics of the OFT wall. The reasons for this change are not clear. Physically, the band constriction locally increases the resistance of blood to flow. This increased blood pressure both upstream and downstream of the band [35] could lead to volume overload and increased myocardial distension. When blood is ejected from the ventricle at high pressure, a pressure wave travels through the OFT. Wave propagation, however, is disrupted by the band creating a site of wave reflection that could delay the forward wave as well. Experimentally, it is observed that the pressure wave travels the OFT faster when there is a band [35]. This observation could also be explained by reasoning that by physically restricting wall motion the band (locally) increases the stiffness of the OFT wall, leading to an increase in wave propagation speed. The relative contributions of passive expansion and active myocardial contraction on OFT motion have not been elucidated. A disruption in conduction of the action potential along the OFT due to the presence of the band (in a way a “foreign object”) cannot be discarded.

### 3.2. OFT Wall Motion: 4D Analysis

**Figure 4:**
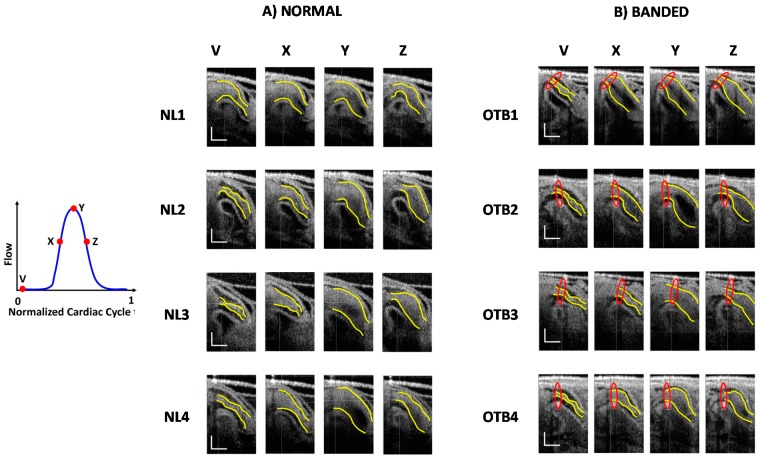
Longitudinal OCT images of the OFT at different phases during the cardiac cycle, with overlaid segmentations of the OFT lumen shown in yellow. The images compare the configurations of (**A**) four normal (NL); and (**B**) four banded (OTB) embryos. The images show the OFT configuration from close to the ventricle (left upper corner of the images) to near the aortic sac (right down corner of the images). Phases depicted (see also the flow schematics on the left) are: (V) closed OFT lumen; (X) midway opening; (Y) maximum flow, fully open configuration; and (Z) midway contraction, for each embryo. The location of the band is shown in red. Scale bars are 250 μm.

To analyze OFT cardiac motion in more detail, we segmented the lumen and myocardium surfaces from 4D OCT images. Due to technical difficulties (e.g., contrast, position), we could not segment the OFT from all the 4D images, but accurate segmentation was achieved for four normal and four banded hearts (see Figure 4). Segmentations spanned the cardiac cycle and thus represented the heart OFT from full lumen closure to wall expansion followed by wall contraction and back to lumen closure.

**Figure 5:**
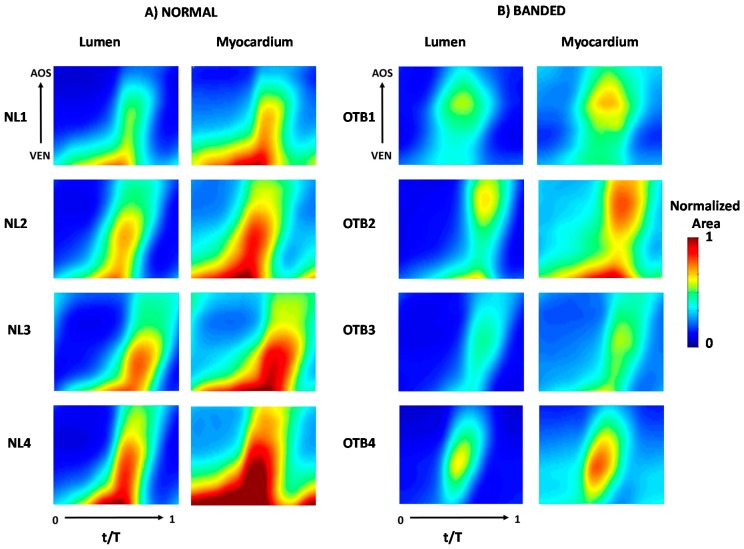
2D area plots of the OFT lumen and myocardium. The plots compare the motion of (**A**) four normal (NL); and (**B**) four banded (OTB) embryos. In each plot, the x-direction (horizontal direction) indicates normalized time over the cardiac cycle, with t time and T cardiac cycle period; the y-direction (vertical direction) indicates normalized length along the segmented portion of the OFT from the section closer to the ventricle (VEN) to the section closer to the aortic sac (AOS), which determines the end of the OFT. Areas are normalized to the maximum lumen area of the normal embryos (occurring for NL4), and, thus, the maroon color features saturation (area > 1) for some myocardium plots. The data shows biological variations among embryos as well as the effects of banding on OFT wall motion. It is evident that the band does not merely constrict the lumen area, but its placement affect cardiac wall motion and mechanics.

The 4D wall motion of the OFT was visualized using 2D area plots (see Figure 5). The OFT wall has a peristaltic-like motion, with lumen cross-sections expanding and contracting sequentially along the OFT tube. This peristaltic motion is the result of interactions with blood flow ejected from the ventricle and active contraction of the OFT myocardium. In 2D area plots, the peristaltic-like motion is evident because maximum (or minimum) areas along the OFT are not synchronized in time, and slightly diagonal expansion/contraction color-bands emerge. In banded embryos wall motion is disrupted (see Figure 5). The inlet of the OFT shows a restricted motion, and hence reduced area, due to the constriction of the band. The OFT, however, expands to a larger area further downstream. Note that some banded embryos show a more synchronous wall expansion/contraction pattern along the OFT length (e.g., in Figure 5 compare NL2, with a clear peristaltic-like motion, with OTB2, with a more simultaneous wall motion). This “synchronous” wall motion pattern in banded embryos is consistent with an increased pressure wave speed along the OFT after banding, which was observed experimentally by measuring blood pressure [35]. Together, these results provide valuable insights into the dynamic motion of normal and banded OFTs as they expand and contract over the cardiac cycle, and how this dynamic motion is affected by the band.

### 3.3. Blood Flow within the OFT: 4D CFD Modeling

Blood flow in the OFT was influenced by curvature, the endocardial cushions, and the changing lumen geometry as the walls of the OFT expanded and contracted. These dynamics were evidenced by examining OCT images [21] and then by analyzing results from our embryo-specific CFD models. As the OFT walls expanded, blood flow rate increased and reached a maximum when OFT walls were most expanded. When the OFT walls contracted, in contrast, blood flow rate decreased until flow stopped. Spatially, blood flow velocities increased towards the OFT outlet due to OFT tapering. In banded hearts, blood flow velocities increased (with respect to control) close to the region of the band, as a result of both mass conservation and the preservation of the stroke volume [21].

**Figure 6:**
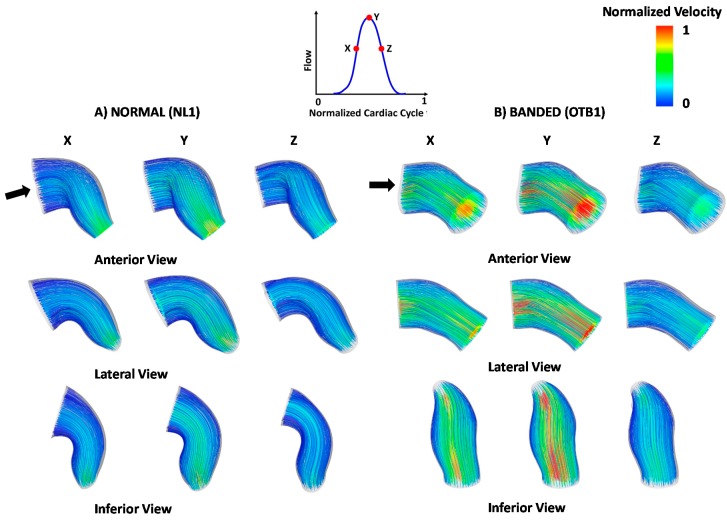
OFT blood flow velocities obtained from CFD simulations. 3D OFT configurations and flow patterns are shown at three times over the cardiac cycle for a representative (**A**) normal and (**B**) banded embryos. Black arrows indicate direction of blood flow. For each embryo, multiple views (anterior, lateral and inferior) of the OFT are shown. The streamlines depict the flow pattern in the OFT, and are colored by velocity magnitude, normalized to the normal embryo maximum velocity, which occurs close to the centerline of the OFT outlet region.

Simulation of blood flow dynamics on a control and a banded heart OFT revealed additional details of the blood flow distribution within the OFT (see Figure 6). There were no evident flow recirculation regions in the normal or banded OFTs, as expected given the negligible flow inertia (Re < 7 and Wo < 0.5, computed from the maximally expanded wall configuration and considering peak velocities). Further, the velocity profiles, while affected by the OFT curvature, remained parabolic-like along the OFT with maximal velocities approximately occurring along the lumen centerline. In the normal embryo, the peak velocity, which occurs at the OFT outlet region, more than doubles the peak velocity in the central portion of the OFT, due to tapering. In the banded OFT, in contrast, peak velocity in the outlet region is similar to that of the normal embryo, but peak velocity in the region of the band is elevated with respect to that of the normal embryo and, for the particular OFT simulated, the peak velocity at the band site is slightly larger than the velocity at the outlet. Computed velocities agree very well with experimental data reported before [21,36,37,38].

**Figure 7:**
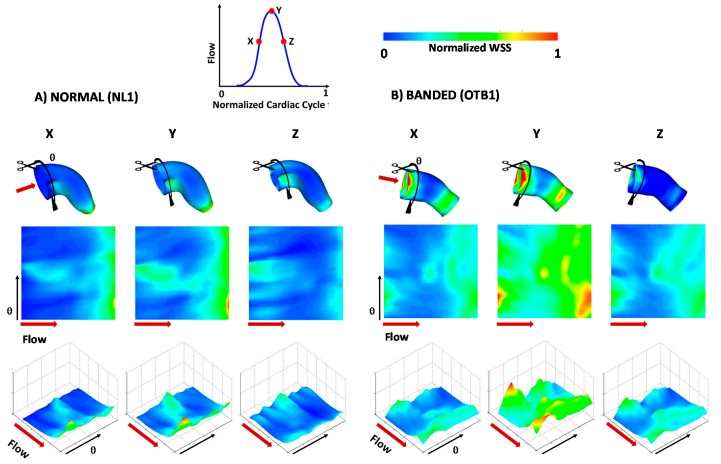
Normalized wall shear stresses (WSS) on the OFT endocardium computed from CFD simulations. The WSS are depicted at three times during the cardiac cycle, for representative (**A**) normal (NL1); and (**B**) banded (OTB1) embryos. To avoid WSS inaccuracies due to boundary effects in the CFD computations, results are shown for the central portion of our OFT models, excluding about 50 µm of the OFT from the inlet and outlet portions. To better visualize the heterogeneity of WSS over the endocardium, we show normalized WSS over three rows of plots. Top row: 3D endocardium surface with WSS color coded on the surface. Middle row: 3D WSS values were mapped to a square section, as if the endocardium was cut as shown in the top 3D picture, and WSS values displayed flat on a plane. Flow direction, from OFT inlet to outlet is shown by a red arrow, and the direction along the OFT circumference (θ) depicted by a black arrow. Bottom row: Depicts WSS as in the middle row, but in addition to color coding the WSS, WSS values are shown by elevation as well, to help visualizing the WSS distribution. WSSs were normalized to the maximum WSS in the normal embryo, which occurred at the OFT outlet region during maximum flow.

Embryo-specific CFD modeling of the OFT enabled quantification of spatial and temporal wall shear stress (WSS) and/or wall shear rates (WSR) distributions for both normal and banded OFTs. WSS and WSR (they are proportional to each other with WSS/WSR = µ, the viscosity of blood) are of critical importance as they contribute to the mechanical load sensed by endocardial cells lining the heart lumen [25,27]. While WSS and WSR in the OFT have been previously obtained directly from measured blood flow velocities using particle tracking methods [38], OCT limitations in acquiring Doppler velocities (wrapping, only one velocity component is measured) prevent direct computations of WSS and WSR. To circumvent these difficulties our approach was to complement OCT data with computational CFD modeling. We analyzed the spatial and temporal WSS distributions on the OFT endocardium, and how banding affects this distribution. For both the normal and banded embryos the distributions of WSS were heterogeneous, and depended on curvature as well as variations in lumen area (see Figure 7).

Maximal WSS was obtained close to peak flow conditions, when the OFT walls were most expanded and blood flow rate was maximal. In the normal embryo, WSS was larger near the inner curvature region due to curvature, and close to the outlet due to tapering. The endocardial cushion near the inner curvature experienced a larger WSS than the outer curvature cushion (see Figure 7 and [24]). In the banded embryo, WSS were larger not only at the outlet region due to tapering but also at the band site due to increased velocities resulting from constrained (smaller) lumen areas from the band placement. Thus, the inlet region of the banded OFT, which approximately corresponded to the banded site (see Figure 4) featured larger WSS than the central portion (see Figure 7) at all times.

While the simulations presented here were performed on one representative normal and one representative banded embryo, because the models were embryo-specific, computations were subjected to biological variations and variations in band tightness. A more thorough comparison of embryos needs to be done before making conclusions about changes in WSS among normal and banded embryos. These computations exemplify what can be expected from such comparison. Spatial and temporal variations of WSS in the OFT likely affect endocardium responses differentially and locally throughout the OFT.

## 4. Conclusions

Cardiac development and hemodynamic conditions are interlinked. The heart starts beating soon after formation of the primitive tubular heart, and thus essential cardiogenesis processes, such as formation of valves and chambers, occur under blood flow conditions. Cardiac function, which determines blood flow, depends on heart development; and heart development depends on blood flow. Thus cardiac development and blood flow cannot be separated, and as a consequence it is difficult to distinguish contributions from genetics or teratogens from those of blood flow in congenital heart disease. Nevertheless, it is well accepted that altered blood flows resulting from cardiovascular defects exacerbate detrimental heart remodeling.

Our results are starting to reveal the intricate interactions between the biomechanics of blood flow and embryonic cardiac tissues. Hemodynamic interventions in embryos do not merely alter blood flow or blood pressure. Necessarily, altering blood flow dynamics also affects cardiac function, and thus, when studying hemodynamic effects, it is also difficult to separate effects from blood pressure, wall shear stress, or tissue strain and stress, as they all change together *in vivo*. It is likely that the conduction system is affected, either directly or by an alteration in its maturation. Like gene networks and signaling pathways, cardiac biomechanics have local and global effects on heart development and function. Currently, however, little is known on how blood flow affects cardiac function, development, maturation and beyond. Interdisciplinary studies aiming at better understanding cardiac biomechanics and how biomechanics affects cardiac development are needed, and can one day enable early interventions for children with congenital heart disease.

## Supplementary Materials

The following are available online at www.mdpi.com/2308-3425/3/1/1/s1, Video S1: Chick embryonic normal beating heart; Video S2: Chick embryonic banded beating heart.
